# The Effects of Nine Compounds on Aldehyde-Oxidase-Related Genes in *Bactrocera dorsalis* (Hendel)

**DOI:** 10.3390/genes15010035

**Published:** 2023-12-25

**Authors:** Yan Zou, Yupeng Chen, Duoduo Wang, Xiaowei Xie, Gen Li, Chunyan Zheng, Jian Wen, Hongai Su, Xin Liu, Ling Zeng, Yongyue Lu, Fengqin Cao

**Affiliations:** 1 School of Tropical Agriculture and Forestry, Hainan University, Haikou 570100, China; 21210904000032@hainanu.edu.cn (Y.Z.); xie-xiaowei@outlook.com (X.X.); arcwenjian@gmail.com (J.W.); 22220951320066@hainanu.edu.cn (X.L.); 2 Department of Entomology, South China Agricultural University, Guangzhou 510642, China; yemanjiuban@outlook.com (Y.C.); ligen@scau.edu.cn (G.L.); chunyanzheng01@scau.edu.cn (C.Z.); 274321765@scau.edu.cn (H.S.); zengling@scau.edu.cn (L.Z.); luyongyue@scau.edu.cn (Y.L.); 3 College of Life Sciences, Zhejiang Normal University, Jinhua 321004, China; duoduo538@zjnu.edu.cn

**Keywords:** *Bactrocera dorsalis*, pest control, aldehyde oxidase gene, molecular docking, potential effective compound

## Abstract

*Bactrocera dorsalis* (Hendel) (Diptera: Tephritidae) (*B. dorsalis*) is an important agricultural, major invasive, and quarantine pest that can cause significant damage to the economic value of the fruit and vegetable industry. Male bait is one of the most effective methods of surveying, monitoring, and controlling *B. dorsalis*. In our study, we constructed cDNA libraries using total RNA extracted independently from the antennae, mouthparts, and thoracic legs of male and female adults and the ovipositors of female adults and screened out four aldehyde-oxidase-related genes (AOX-related), *C58800*, *C66700*, *C67485*, and *C67698*. Molecular docking predictions showed that eight compounds, including 3,4-dimethoxycinnamyl alcohol, 3,4-dimethoxy-cinnamaldehyde, deet, ethyl N-acetyl-N-butyl-β-alaninate, n-butyl butyrate, n-butyl butyrate, ethyl butyrate, methyl eugenol, and ethyl acetate, could combine with proteins encoded by the four *B. dorsalis* AOX-related genes. Furthermore, QPCR was performed to confirm that four compounds, including 3,4-dimethoxy cinnamic aldehyde, butyl levulinic acid ethyl ester (mosquito repellent), butyl butyrate, and methyl eugenol, induced significant changes in the AOX-related genes of *B. dorsalis*. These results provide useful information and guidance for the batch screening of potentially useful compounds and the search for effective attractants of *B. dorsalis*.

## 1. Introduction

*B. dorsalis* is considered to be a highly invasive and destructive agricultural pest due to its high dispersal and adaptive capacity [1,2].

Olfactory sensilla are mainly distributed in the antennae of insects, as well as in the wings, feet, genitalia, and auxiliary organs of the head, such as chin and lower lip whiskers. The antennae are the main organs used for sensing odorous substances [3,4]. The process of insect odour recognition is complex. At the level of peripheral nerve sensation, many proteins are involved in this process, including odorant binding proteins (OBPs), olfactory receptors (ORs), odour-degrading enzymes (ODEs), and sensory neuron membrane proteins (SNMPs) [5,6]. Aldehyde oxidase (AOX) is a subfamily of the molybdo-flavoenzyme family (MFE), and the classification number of AOX is EC 1.2.3.1. [7]. AOX is widely present in organisms and participates in a variety of physiological metabolisms. It converts aldehydes formed in the body into non-toxic acids to reduce the toxic effects of aldehydes on the body and participates in intracellular electron transfer; it also plays an important role in physiological activities related to metabolism and reproduction [8,9,10]. AOX has a diverse range of physiological and biochemical functions and can degrade odour molecules in the insect olfactory system. For example, *Culex quinquefasciatus* AOX may be related to insecticide resistance [11].

Attractants are an important tool in the control of *B. dorsalis*. A major attractant at present is methyl eugenol, which can be converted into 2-allyl-4,5-dimethoxy phenol (DMP) and (E) coniferyl alcohol (E-cf) in *B. dorsalis*. DMP is more attractive to male *B. dorsalis* than E-cf and does not pose a risk to human health and the environment [12]. Methyleugenol (ME) can stimulate the odour receptors 63a-1 (OR63a-1) and OR88a in the antennae of mature male *B. dorsalis* [13]. However, at present, few studies on the interaction between compounds and the aldehyde-oxidase-related genes of *B. dorsalis* have been reported.

In this study, transcriptome sequencing was performed on different parts of *B. dorsalis*, and molecular docking and QPCR were used to identify the relationship between *B. dorsalis* AOX-related genes and potential compounds. Finally, it was found that nine compounds, including maldehyde, deet, ethyl acetyl-N-butyl-β-alaninate, n-butyl butyrate, ethyl butyrate, methyl eugenol, ethyl acetate, and sex pheromones, could significantly affect the expression of AOX-related genes in *B. dorsalis* nymphs. These results provide a methodological reference for the batch screening of potentially useful compounds and the search for effective attractants of *B. dorsalis*.

## 2. Materials and Methods

### 2.1. Materials

Insect rearing: *B. dorsalis* was collected from South China Agricultural University Fruit Tree Plantation, Guangdong Province, China and reared as previously described in the South China Agricultural University (Guangzhou, China). The larvae of the *B. dorsalis* colony were provided with an artificial larval diet mixture of torula yeast, wheat powerbran, sugar, sodium benzoate, nipagin, and water in fixed proportions. The adult flies were reared in a cage (60 × 60 × 60 cm) under pesticide-free conditions at 27 ± 1 °C, 70 ± 5% relative humidity, and a photoperiod of 16:8 (L:D) h and provided with water and a mixture of torula yeast and sugar at a fixed rate [14].

CFQ1: 3,4-dimethoxycinnamyl alcohol (CAS: 40918-90-9, HPLC: 97%, BioBioPha Co., Ltd., Yunnan, China); CFQ2: 3,4-dimethoxy-cinnamaldehyde (CAS: 4497-40-9, HPLC: 97%, BioBioPha); CFQ3: ethyl-N-acetyl-N-butyl-β-alaninate (CAS: 52304-36-6, HPLC: 97%, Qingdao Yousuo Chemistry Technology Co., Ltd., Qingdao, China); CFQ4: N-butyl butyrate (CAS: 109-21-7, HPLC: 99%, Sigma–Aldrich, Shanghai, China); CFQ5: ethyl butyrate (CAS: 105-54-4, HPLC: 99%, Sigma–Aldrich); CFQ6: Methyleugenol (CAS: 93-15-2, HPLC: 95%, Sigma–Aldrich); CFQ7: ethyl acetate (CAS: 141-78-6, HPLC: 99%, Tianjin Damao Chemical Reagent Co., Ltd., Tianjin, China.); CFQ8: deet (CAS: 134-62-3, HPLC: 97%, Qingdao Yousuo Chemistry Technology Co., Ltd., Qingdao, China); sex lures (HPLC: ≥98%, Guangzhou Ruifeng Biotechnology Co., Ltd., Guangzhou, China).

Experimental instruments: We used a Mastercycler Pro S gradient PCR instrument (Eppendorf Co., Ltd., Hamburg, Germany), BioradGelDoc XR gel imaging system (Bio-Rad, Hercules, CA, USA), and quantitative PCR instrument (Mx3005p, Agilent Technologies Inc., Santa Clara, CA, USA). A trace sample total RNA extraction kit was used (DP420, TIANGEN BIOTECH Co., Ltd., Beijing, China), as well as a Quant cDNA first strand synthesis kit (KR103, TIANGEN BIOTECH Co., Ltd., Beijing, China).

### 2.2. Generated cDNA Libraries Using Male and Female Adult Antennae, Mouthparts, Thoracic Legs, and Female Ovipositors

We sampled adult male and female antennae, mouthparts, and thoracic legs and female ovipositors. To minimise RNA degradation and sample contamination, these sampling steps were performed on an ultra-clean bench and on ice. The cutting tools, scalpel, and forceps were sterilised. The antennae, mouthparts, thoracic legs, and ovipositors were quickly cut off and placed in tissue lysate (trace sample total RNA extraction kit, DP420, Tiangen Biotech Co., Ltd., Beijing, China), and the antennae, mouthparts, thoracic legs, and ovipositors of each group of 200 flies were treated as one sample replicate. The samples were sent to GeneDenovo (Guangzhou, China) for library construction and genome sequencing using a combination of next-generation sequencing (NGS) on the Hiseq 2500 and third-generation sequencing on the Pacbio RSII. BLAST [15] software was used to compare the Unigene sequence with NR, Swiss-Prot [16], GO [17], COG [18], KOG [19], KEGG [20], using KOBAS2.0 [21]. 

Using transcriptome data to detect gene expression has high sensitivity. Generally, the FPKM value of the protein coding gene expression level that can be sequenced spans six orders of magnitude from 10^−2^ to 10^−4^ [22]. Using the box diagram of FPKM distribution, the dispersion degree of horizontal distribution of gene expression in a single sample was evaluated, and the overall gene expression abundance of different samples was compared.

### 2.3. Comparative Analysis of the Expression of Genes between the Male and Female Parts of B. dorsalis

Based on our independent comparison between female and male *B. dorsalis*, we analysed the gene expression differences between different parts of the female and between different parts of the male and considered the |log2FC| > 1 as a significant difference. A differential expression analysis between sample groups was performed using DESeq (version 4.2.2). At the same time, in the process of differential expression analysis, the significant *p*-value obtained from the original hypothesis testing was corrected using the generally accepted and effective Benjamini–Hochberg method, and the corrected *p*-value, i.e., FDR (false discovery rate), was finally adopted as a key indicator for differential expression gene screening to reduce the false positives caused by independent statistical hypothesis testing of the expression values of a large number of genes.

### 2.4. Screening and Identification of AOX-Related Genes in B. dorsalis

Based on the comparative analysis of gene expression between the male and female parts of *B. dorsalis*, the keywords “dehydrogenase” and “aldehyde oxidase” and “aldehyde” were selected from the gene databases GO [23], KEGG [24], Pfam [14], KOG ((http://ftp.ncbi.nih.gov/pub/COG/KOG/kyva, accessed on 1 December 2022), swissprot https://www.expasy.org/resources/uniprotkb-swiss-prot, accessed on 9 December 2022), and NR (https://ftp.ncbi.nlm.nih.gov/blast/db/, accessed on 19 December 2022). The ORF of the screened gene was predicted using the DNAStar software package (version 7.1, DNAstar Inc.). Using the GenBank database from the NCBI (https://www.ncbi.nlm.nih.gov/, accessed on 10 December 2022), the homologous sequences were compared and analysed using the BLASTX method.Clustal X (version 1.81) was used for multiple sequence alignment of the sequences downloaded from NCBI. The phylogeny and molecular evolution were analysed using the neighbour-joining method with MEGA (version 11.0.11).

Both AOX and XDH are proteins in the MFE, and the structural features of AOX and XDH were similar. The genes identified as AOX and XDH with similar sequences were named AOX-related genes.

### 2.5. Docking Simulations

Four protein structures corresponding to the four AOX-related genes *C58800*, *C66700*, *C67485*, and *C67698* were found in the Swissmodel database (https://swissmodel.expasy.org/interactive, accessed on 8 June 2023), while the the 3D structures of eight components were sourced from the PubChem database ((https://pubchem.ncbi.nlm.nih.gov/, accessed on 8 June 2023) and the protein structures of the eight components selected for this study from AutoDock-vina. The 3D structures of the eight components CFQ1~CFQ8 were obtained from the PubChem database (https://pubchem.ncbi.nlm.nih.gov/, accessed on 8 June 2023). PYMOL (version 2.5.4) and LIGPLOT (version 2.2.5) software were used for image visualisation.

### 2.6. Changes in B. dorsalis AOX-Related Genes Expression under the Action of 9 Compounds

Four protein structures corresponding to the four AOX-related genes *C58800*, *C66700*, *C67485*, and *C67698* were found in the Swissmodel database. The prediction and docking simulations showed that the compounds of (3,4-dimethoxycinnamyl alcohol, 3,4-dimethoxy-cinnamaldehyde, Deet, ethyl N-acetyl-N-butyl-β-alaninate, n-butyl butyrate, ethyl butyrate, methyl eugenol and ethyl acetate) were able to bind to 4 protein structures. We selected the 3rd-day pupae of *B. dorsalis* to perform the experiment. Chemical concentrations of 20 μg/μL were used [12]. Three replicates were made for each chemical treatment and 500 normal pupae were used in each set of replicates; then, 1 mL of the chemical was placed in a culture plate with cotton, and the culture plate was placed in a 30*30*35 cm transparent and breathable acrylic box where the pupae were kept, and the sterile water treatment was used as a control. We collected *B. dorsalis* antennae, ovipositors, thoracic legs, and mouthparts from insects that had fledged 24 h after treatment for RNA extraction, reverse transcription, and QPCR. The compound-treated transcriptome was further analysed with the untreated transcriptome. Four AOX-related genes, *C58800*, *C66700*, *C67485*, and *C67698,* were analysed for differential changes after different compound treatments.

The QPCR primers were designed as follows: *C58800* (F: GGACTACCCGAGGTCCATCT, R: GGTGAGACTTGGCGACAGA), *C66700* (F: TATCGTTCGCCGACGCATTA, R: AGAACCGTACATGGGCTG), *C67485* (F: GGTCCATGTCGTTGGGCTTA, R: AGGATCCATTGGCAGCGAAA), and *C67698* (F: CACCGTACACATCAACCCCA, R: ATCTGTTTGCCCGCGAAGTA).

## 3. Results

### 3.1. Generation of cDNA Libraries Using Male and Female Adult Antennae, Mouthparts, and Thoracic Legs and Female Ovipositors

The clean data obtained totalled 77.52 Gb, and the base percentage of Q30 was not less than 96.11% (Supplementary Table S1, Supplementary Figure S1B). After assembling and splicing, 77,765 pieces of Unigene were obtained, of which the GC content was 42.33%. The N50 of Unigene was 1482 bp, with the longest Unigene being 2000 bp, the shortest 200–300 bp, and the average length 938.41 bp. The matching rate was over 80%. In addition, the position distribution of mapped reads of each sample was obtained by evaluating the random degree of mRNA fragmentation (Supplementary Figure S1C). Using the box diagram of FPKM distribution, the main area of log10(FPKM) was found to be −1~1. This shows that the assembly integrity is high (Supplementary Figure S1D).

### 3.2. Comparative Analysis of Genes between Male and Female Parts of B. dorsalis

**Figure 1:**
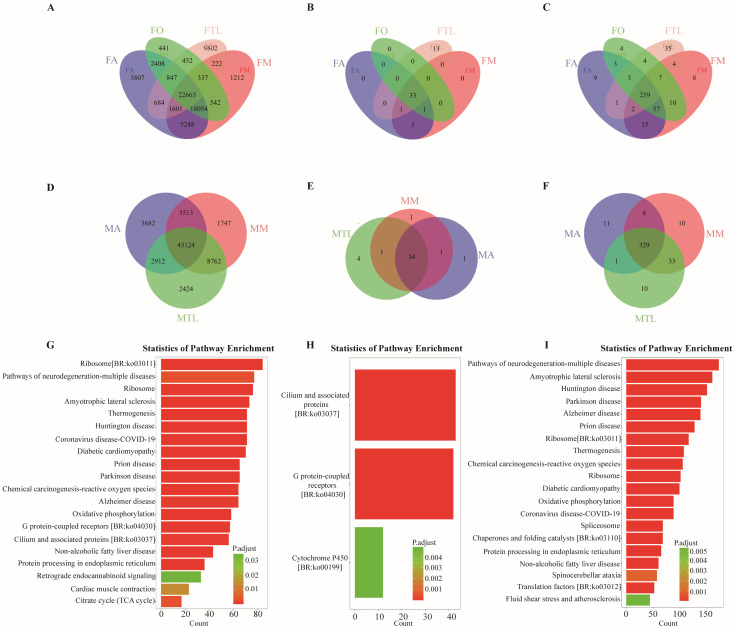
Comparative analysis of genes between male and female parts of *B. dorsalis*. FA: female antennae; FO: female ovipositors; FTL: female thoracic legs; FM: female mouthparts; MA: male antennae; MM: male mouthparts; MTL: male thoracic legs. (**A**) Comparative analysis of genes among all parts of female *B. dorsalis*; (**B**) KEGG enrichment of genes in different parts of female *B. dorsalis*.; (**C**) GO terms of genes from different parts of *B. dorsalis* females; (**D**) comparative analysis of genes among different parts of male *B. dorsalis*; (**E**) KEGG analysis of genes among all parts of *B. dorsalis* males; (**F**) GO enrichment analysis among all parts of *B. dorsalis* males; (**G**) FA vs. FM: the differential gene KEGG between female antennae and female mouthparts was enriched; (**H**) FA vs. FO: the differential gene KEGG between female antennae and female ovipositor was enriched; (**I**) FA vs. FTL, KEGG, a differential gene between female antennae and female thoracic leg, is enriched.

By comparing the genes in different tissues of individual *B. dorsalis*, we found that the female thoracic leg contained a total of 9802 genes that were differentially expressed in male and female thoracic legs, which was the highest number compared with DEGs in antennae, ovipositors, and mouthparts (Figure 1A,B). 

Based on the independent comparison between female and male *B. dorsalis*, gene expression between different parts of the female and male was compared. Differential expression analysis between sample groups was performed using DESeq. In terms of the number of GO terms, female thoracic legs had 35 more than female antenna, female ovipositor, and female mouthparts; male antenna had 11 more than male thoracic legs and male mouthparts (Figure 1C,F). Regarding the KEGG pathways, female thoracic legs had the highest number of KEGG pathways compared to female antennae, female ovipositors, and female mouthparts. Male thoracic legs had the highest number of pathways compared to male antennae and male mouthparts at 4 (Figure 1B,E). 

KEGG and GO enrichment analysis of the genes in different tissues of individual *B. dorsalis* revealed that among the 33 pathways of the thoracic leg, AOX-related genes were found (Figure 1I). KEGG enrichment showed that the differences in KEGG analysis were more pronounced in females than in males, with the number of female thoracic leg KEGG differences higher than in male thoracic legs (Figure 1B,E). The differential genes were mainly enriched in the pathways of multiple neurodegenerative diseases, including amyotrophic lateral sclerosis, Huntington’s disease, Parkinson’s disease, Alzheimer’s disease, etc.

### 3.3. Identification of AOX-Related Genes in B. dorsalis

**Figure 2:**
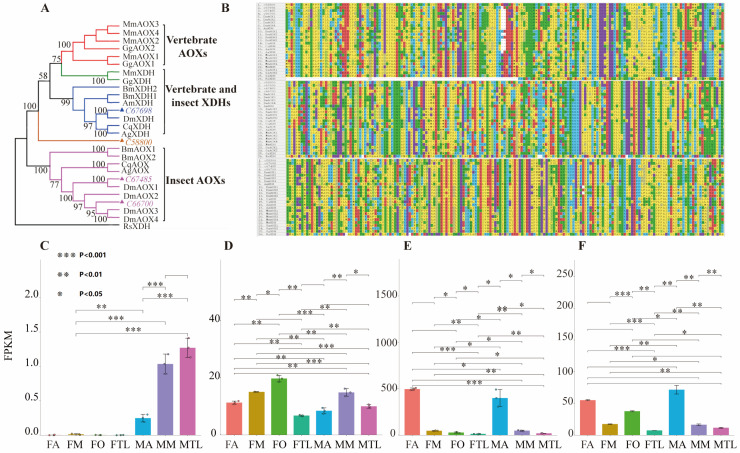
Identification of AOX-related genes in *B. dorsalis*. (**A**) Phylogenetic tree analysis of the predicted four degrading enzyme genes *C58800*, *C67698*, *C66700*, and *C67485* with other species’ AOXs and XDHs, and the sequence information and NCBI GenBank login number used are as follows: Drosophila melanogaster (DmAOX1, NP_650475; DmAOX2, NP_732047; DmAOX3, NP_650477; DmAOX4, NP_650478; DmXDH, NP_524337); Apis mellifera (AmXDH, XP_016768886). Bombyx mori (BmAOX1, NP_001103812; BmAOX2, NP_001103811; BmXDH1, NP_001037325; BmXDH2, NP_001037333); Culex quinquefasciatus (CqAOX, XP_038118267; CqXDH, XP_038112406); Anopheles gambiae (AgAOX, XP_316291; AgXDH, AAO14865); Mus musculus (MmAOX1, NP_033806; MmAOX2, NP_001008419; MAOX3, NP_076106; MmAOX4, NP_076120; MmXDH, NP_035853); Gallus gallus (GgAOX1, NP_046777290; GgAOX2,NP_001034690; GgXDH, NP_990458); Rhodobacter sphaeroides (RsXDH, ANS35864). (**B**) *C58800*, *C67698*, *C66700*, and *C67485* amino acid sequences were compared with multiple sequences of other insect homologous genes; (**C**–**F**), respectively, correspond to the expression levels of genes *C58800*, C67698, C66700, and C67485 in antennae, thoracic legs, mouthparts, and ovipositors of B. dorsalis. Asterisks above the error bars indicate the significant difference analyzed (*p* < 0.05).

Based on *B. dorsalis* transcriptome annotation, four AOX-related genes were found, including *C58800*, *C67698*, *C66700*, and *C67485* (Supplementary Table S2). According to the evolutionary tree and sequence comparison, they were predicted to be AOX and xanthine dehydrogenase (XDH) (Figure 2A). The sequences of these four genes were analysed. The size of *C58800* was 2886 bp, with an open reading frame 2487 bp in length encoding 828 amino acids. The size of *C67698* was 5463 bp, with an open reading frame of 3552 bp, encoding 1183 amino acids. The size of *C66700* was 3989 bp, and the maximum open reading frame was 3792 bp, encoding 1263 amino acids. The size of *C67485* was 4687 bp, and the maximum open reading frame was 4074 bp, encoding 1357 amino acids (Figure 2B).

AOX is usually composed of a homodimer formed by two identical subunits, and each subunit has two [2Fe-2S] redox centres, a flavin cofactor (FAD), and a substrate-binding domain. By comparing and analysing the sequences of these four genes, two conserved iron–sulphur (2Fe-2S) redox reaction centres were found in the N segment and eight conserved cysteine residues were found in this centre. However, the domains were not detected in *C66700* and *C58800*. The N-terminal is connected to the FAD− binding site through a less conserved region. XDH has a typical conservative NAD+ binding site in the FAD− binding site region, but AOX does not contain this site, while *C67698* has this site. The phylogenetic tree was constructed with the four enzymes and a number of AOXs and XDHs from other species using the neighbour-joining method. The results show that *C66700* and *C67485* were clustered with AOXs of other species, and *C67698* was clustered with XDH. Through sequence analysis, it was speculated that *C66700* and *C67485* encodes AOX in *B. dorsalis* and *C67698* encodes XDH. Through NCBI comparison analysis, it was speculated that the gene *C57605* might be lactate dehydrogenase (Figure 2A). The four identified genes, C66700, C6748, C67698, and C57605, were classified as AOX-related genes.

The FPKM of the four genes related to AOX, including *C58800*, *C67698*, *C66700*, and *C67485* in thoracic legs, antennae, mouthparts, and ovipositors from the transcriptome are shown in Figure 2. It was found that the expression level of *C58800* was higher in the olfactory tissues of males than that of females. The expression of *C58800* was the highest in the male thoracic leg, which suggested that the gene *C58800* is mainly concentrated in the olfactory tissues of males. The genes *C67698*, *C66700*, and *C67485* were all expressed in the olfactory tissues of *B. dorsalis*. Among them, the expression level of *C67698* in the thoracic legs, antennae, and mouthparts of females was higher than that of males, and the genes *C67700* and *C67485* were mainly expressed in the antennae of males and females. The genes *C58800*, *C67698*, and *C67485* were all highly expressed in male antennae (Figure 2C,E,F), and it was assumed that the three AOX-related genes have a greater effect on olfaction in males.

### 3.4. Docking Simulations

**Figure 3:**
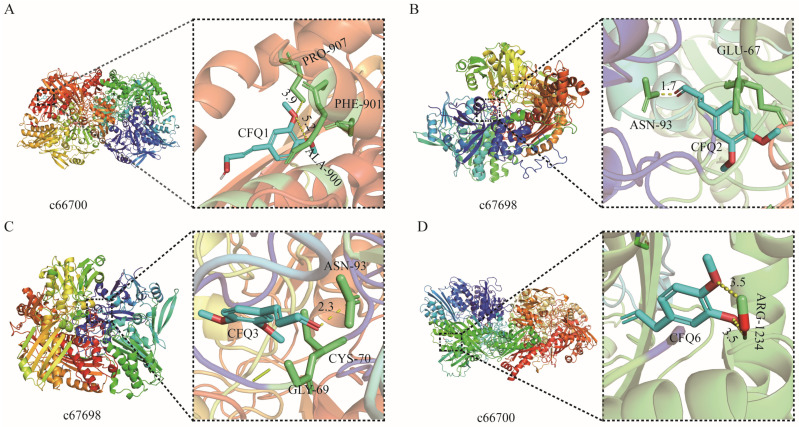
Visual diagram for protein docking of eight components with *B. dorsalis*. (**A**–**D**) are the docking diagrams of components CFQ1, CFQ2, CFQ3, and CFQ6, respectively.Colorful cartoon patterns are protein, the blue stick structure is chemical composition. Yellow dotted lines are linked hydrogen bonds.

In order to test the effects of eight compounds (CFQ1–8) on the AOX-related genes in *B. dorsalis* and whether the components of the eight reagents have effective binding targets, molecular docking was performed between the 3D conformation of eight components and four proteins of the four AOX-related genes. A total of 288 binding relationships were detected (Supplementary Table S3). The lower the affinity value, the more stable the binding between protein and ligand, and the better the binding in protein–ligand interaction. An affinity ≤ −5 kcal/mol was considered to have good binding [25]. According to the binding affinity value, the binding relationships of five components, CFQ1, CFQ2, CFQ3, CFQ6, and CFQ8, were simulated to bind four proteins, among which four proteins had an affinity equal to or lower than −5 kcal/mol, and it was confirmed that these four components can effectively target the proteins involved in the complex pathway of *B. dorsalis* (Figure 3). Component CFQ6 can bind three genes and three proteins with an average binding affinity of −5.49 kcal/mol. It can bind best with protein structure c66700 responding to gene *c66700* with a binding affinity of −6.56 kcal/mol. Components CFQ1, CFQ2, CFQ3, and CFQ8 could bind four genes and four proteins with average binding affinities of −5.42, −5.45, −5.45, and −5.55 kcal/mol. CFQ2 and CFQ3 can bind best with protein structure c67698 responding to gene *c67698* with a binding affinity of −6.32 kcal/mol. These five components are thought to affect the olfactory genes of *B. dorsalis* and are associated with the four predicted AOX-related genes.

### 3.5. Changes in B. dorsalis AOX-Related Gene Expression by Nine Compounds

**Figure 4:**
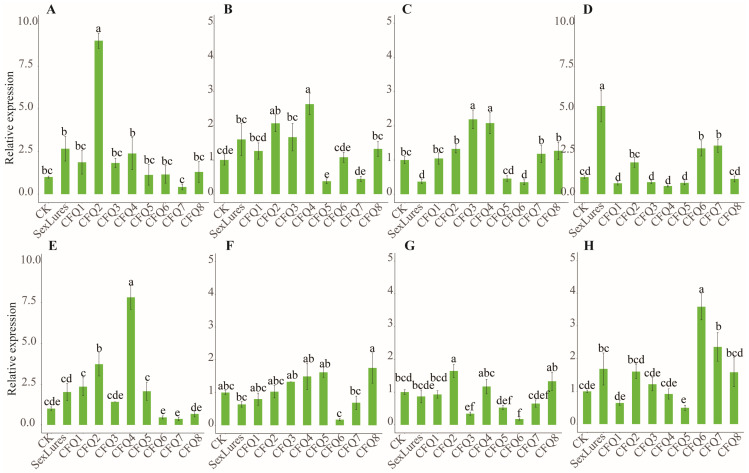
Expression changes in AOX-related genes in *B. dorsalis* under the action of nine components. (**A**–**D**) correspond to female AOX-related genes; (**E**–**H**) correspond to the expression changes in the male AOX-related genes *C58800*, *C67698*, *C66700*, and *C67485* under the action of nine components. Different letters above the error bars indicate the significant differences analyzed (*p* < 0.05).

In determining the influence of nine compounds on the AOX-related genes in *B. dorsalis*, compared with deionised water (CK) and sex lures, all nine compounds were found to alter the expression of four AOX-related genes, with CFQ2 significantly upregulating the expression of four genes in females and males, especially *C58800*. CFQ6 and CFQ7 significantly downregulated the expression of *C67698* in females. It was speculated that the drugs CFQ6 and CFQ7 might have a behavioural effect on *B. dorsali* females (Figure 4A–D). The females were more significantly upregulated than the males (Figure 4E,F). The expression of genes *C58800* and *C67698* in females was significantly upregulated by CFQ6, while their expression was significantly downregulated in males. CFQ4 significantly upregulated the expression of the *C5880* gene in males and CFQ6 significantly upregulated the expression of the *C67485* gene in males. CFQ2 and CFQ4 and sex lures significantly upregulated C5880 gene expression in both sexes. It was hypothesised that CFQ2 and CFQ4 and sex lures had the same priming effect on *B. dorsali*. (Figure 4A,E). The above results are consistent with the binding reaction between the components and the AOX-related genes predicted by the docking model. Molecular docking can be used to screen components related to *B. dorsalis* control.

### 3.6. Predicted Mechanism of the B. dorsalis AOX-Related Genes

**Figure 5:**
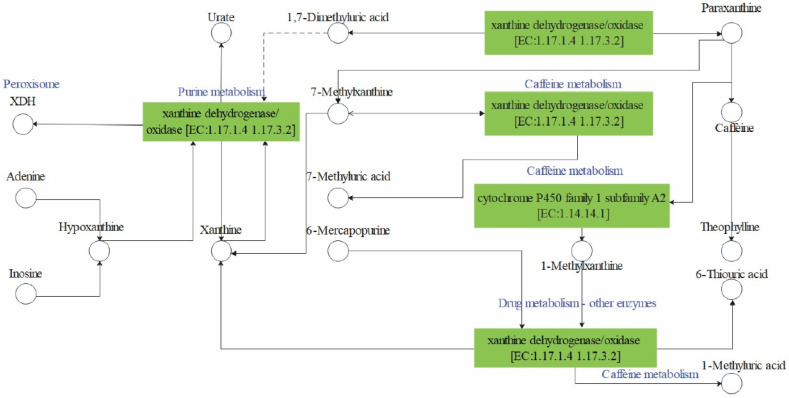
Predicted mechanism of action of the *B. dorsalis* AOX-related gene.

KEGG enrichment analysis suggested that the AOX-related genes, including *C58800*, *C66700*, *C67485*, and *C67698*, were mainly involved in four pathways: ko00230, ko00232, ko00983, and ko04146. These AOX-related genes were involved in the same pathways after treatment with nine components. The pathways in which the four genes were mainly involved are shown in Table 1. Four pathways were integrated into one pathway (Figure 5).

All four genes were involved in purine metabolism (ko00230), caffeine metabolism (ko00232), drug metabolism of other enzymes (Ko00983), and peroxisome pathways (ko04146).

The four genes in the ko00230 pathway were all involved in the transformation from urea to xanthine [EC: 1.17.1.4 1.17.3.2] and from hypoxanthine to xanthine [EC: 1.17.1.4 1.17.3.2], and the corresponding enzymes were xanthine dehydrogenase/oxidase. Four genes in the ko00232 pathway were involved in the conversion of 7-methylxanthine to 1,7-dimethyluric acid [EC:1.17.3.2], 7-methylxanthine to 7- methylxanthine acid [EC:1.17.3.2], and theobromine to 3,7-dimethyluric acid [EC: 1.17.2]. Four genes in the ko00983 pathway were involved in the synthesis process from 6-mercaptopurine (prodrug) to 1-methyl-4-nitro-imidazole [EC: 1.17.3.2]. All four genes were involved in the metabolism of xanthine dehydrogenase XDH. The ko00230 pathway (purine metabolism pathway) mentioned above has been reported to be closely related to the feeding behaviour of Leishmania and other insects [26,27]. We combined the four pathways, ko00230, ko00232, ko00983, and ko04146, into one composite pathway. AOX-related genes including *C58800*, *C66700*, *C67485*, and *C67698* might be involved in the ko00230 pathway, which can convert urea to xanthine [EC: 1.17.1.4 1.17.3.2] and convert hypoxanthine to xanthine [EC: 1.17.1.4 1.17.3.2]. CYP450 was involved in the pathway [EC: 1.17.1.4 1.17.3.2]. Cytochrome is metabolized by ko00232 (caffeine metabolism). It has been suggested that the four genes *C58800*, *C66700*, *C67485*, and *C67698* might affect the behaviour of *B. dorsalis* by affecting xanthine dehydrogenase/oxidase and other enzyme pathways of drug metabolism and purine metabolism in the composite pathway. Through enrichment annotation, we hypothesise that these genes, *C58800*, *C66700*, *C67485*, and *C67698*, affect the behaviour of *B. dorsalis* mainly by affecting xanthine dehydrogenase/oxidase and other enzyme pathways of drug metabolism and thus ultimately purine metabolism.

## 4. Discussion

In this study, cDNA libraries were generated using male and female adult antennae, mouthparts, and thoracic legs and female ovipositors. A transcriptome analysis resulted in 77,765 unigenes, and the AOX-related genes were screened. Molecular docking prediction and further QPCR verification showed that eight components had a functional relationship with *B. dorsalis* AOX-related genes. This study provides a reference for batch mining of potentially useful components of *B. dorsalis*.

Four genes related to AOX, *C58800*, *C67698*, *C66700*, and *C67485*, were predicted. It has been reported that the genes encoding AOX were cloned from *silkworm Silkworm*, *Mamestra brassicae*, *Amyeloistransitella*, and other insects [28]. By mining the genome and transcriptome data of many insects, a large number of AOX genes were identified, many of which are specifically or highly expressed in insect antennae, indicating that they may be involved in the degradation of aldehyde odorants [29,30,31,32]. The results in this study also proved that AOX-related genes are significantly affected by the eight compounds that were selected with our methods, including the use of the transcriptome, docking predictions, and QPCR. These results further indicate that AOX-related genes play an important role in the chemical perception of insects and are worth studying further. 

The four genes associated with AOX in females had a greater impact on the female thoracic leg than external stimuli. Interestingly, in the identification of AOX-related genes in *B. dorsalis*, we found that the genes *C58800*, *C67698*, and C67485 all showed significant expression in male antennae. *C66700* expression in male antennae was not significant (Figure 2C–F). 

Among them, 3,4-Dimethoxy-cinnamaldehyde is an aldehyde, N-butyl butyrate and ethyl acetate are esters, and ethyl butyrate is a short-chain ester. Methyl eugenol is a phenylpropane-like compound, Deet is an amide, 3,4-Dimethoxycinnamyl alcohol is an aromatic, and Ethyl-N-acetyl-N-butyl---alaninate has a repellent effect. Methyl eugenol in these compounds has long been proven to have an attractive effect on *B. dorsalis* males, which can excite the odour receptors OR63A-1 and OR88A in the antennae of mature male *B. dorsalis* [13]. This attractive effect can be enhanced by adding ethyl acetate into the food-borne attractant of *B. dorsalis*. *Adelphocoris fasciaticollis* can be attracted by n-butyl butyrate, and *B. dorsalis* will be metabolized into 3,4-dimethoxycinnamyl alcohol in the rectum after eating ME These compounds and our methods of discovering the potential compounds can provide a meaningful reference for the control of *B. dorsalis*.

## Figures and Tables

**Table 1:** Pathways involved by four genes from *B. dorsalis*.

Name	ID	Pathway
C58800, C66700, C67485, C67698	ko00230	Purine metabolism
C58800, C66700, C67485, C67698	ko00232	Caffeine metabolism
C58800, C66700, C67485, C67698	ko00983	Drug metabolism—other enzymes
C58800, C66700, C67485, C67698	ko04146	Peroxisome

## Data Availability

Not applicable.

## Supplementary Materials

The following supporting information can be downloaded at: https://www.mdpi.com/article/10.3390/genes15010035/s1. Figure S1: Construction of *Bactrocera dorsalis* transcriptome. (A) Parts needed for the establishment of gene libraries of female and male *B. dorsalis*; (B) The Figure of length statistics of *B. dorsalis* Unigene; the horizontal coordinate represents the length of the UniGene interval; the longitudinal coordinate indicates the number of UniGene; (C) The position distribution of reads mapped on mRNA in each sample; the horizontal coordinate is mRNA position, and the vertical coordinate is the percentage of reads in reads mapped; (D) The FPKM boxplots of every sample; the horizontal coordinates represent the samples, and the longitudinal coordinates represent the numerical values of the fpkm; Table S1: The result of transcriptome construction; Table S2: CDS of genes C58800, C67698, C66700, and C67485; Table S3: Docking results of 8 components with 4 proteins; Table S4: Gene expression of females; Table S5: Gene expression of males.
