# Supplementary material for: The Effects of Nine Compounds on Aldehyde-Oxidase-Related Genes in Bactrocera dorsalis (Hendel)
Source: Genes (Basel). 2023 Dec 25;15(1):35. doi: 10.3390/genes15010035 (PMC10815873; doi:10.3390/genes15010035)
Supplement: Supplementary file 1 [file genes-15-00035-s001.zip › Supplementary/Supplementary Table S1_The result of Transcriptome construction.docx]

Table S1a The statistics of assembly result for *B. dorsalis*

| Number | N50 | Max lengt（bp） | Min lengt（bp） | Mean Length（bp） |
| --- | --- | --- | --- | --- |
| 77，765 | 1,482 | 2000^+^ | 200 - 300 | 938.41 |

N50：The length of Unigene N50：Mean Length：The average length of Unigene

Table S1b Summary of transcriptome sequencing for different organs of *B. dorsalis*

| **Samples** | **Clean Reads** | **CleanData** | **（GC Content）** | **%≥Q30** | **Mapped Ratio** |
| --- | --- | --- | --- | --- | --- |
| female antennae | 24,756,430 | 6,162,813,302 | 44.00% | 96.46% | 82.21% |
| female antennae | 28,403,853 | 7,077,110,830 | 43.59% | 96.53% | 81.48% |
| female mouthpart | 25,892,115 | 6,457,331,664 | 44.10% | 96.49% | 83.67% |
| female mouthpart | 26,578,114 | 6,628,924,788 | 44.33% | 96.35% | 84.50% |
| female leg | 21,286,986 | 5,306,266,814 | 43.82% | 96.90% | 84.76% |
| female leg | 19,525,522 | 4,869,090,282 | 44.87% | 96.84% | 84.02% |
| female ovipositor | 21,449,421 | 5,344,956,366 | 44.09% | 96.69% | 83.56% |
| female ovipositor | 19,727,088 | 4,919,708,406 | 44.02% | 96.72% | 85.81% |
| male antennae | 19,229,750 | 4,802,905,366 | 41.43% | 96.54% | 88.86% |
| male antennae | 16,165,338 | 4,038,303,496 | 41.57% | 96.43% | 87.08% |
| male mouthpart | 22,663,800 | 5,646,803,200 | 44.26% | 96.27% | 83.53% |
| male mouthpart | 21,370,205 | 5,321,265,932 | 45.44% | 96.45% | 84.00% |
| male leg | 23,346,191 | 5,800,624,134 | 46.05% | 96.17% | 81.95% |
| male leg | 20,687,299 | 5,147,162,982 | 45.69% | 96.11% | 82.54% |

Table S1c Annotation of Unigenes

| **Anno_Database** | **Annotated_Number** | **300<=length<1000** | **length>=1000** |
| --- | --- | --- | --- |
| COG_Annotation | 15430 | 7467 | 6864 |
| GO_Annotation | 18353 | 7663 | 9092 |
| KEGG_Annotation | 16665 | 7840 | 7473 |
| KOG_Annotation | 29663 | 14709 | 11753 |
| Pfam_Annotation | 32654 | 16036 | 14257 |
| Swissprot_Annotation | 20889 | 9221 | 10203 |
| nr_Annotation | 35250 | 16437 | 15419 |
| All_Annotated | 44123 | 22419 | 16718 |

Table S1d The number of genes in each part of *B. dorsalis*

| **Full name of position** | **Gene dosage（FPKM＞0）** | |
| --- | --- | --- |
|  | Female | Male |
| Antenna | 55312 | 53231 |
| mouthparts | 49879 | 57146 |
| Male，Thoracic leg | 36608 | 57222 |
| Female，Ovipositor | 45744 |  |
