# Supplementary figures and images for: The Effects of Nine Compounds on Aldehyde-Oxidase-Related Genes in Bactrocera dorsalis (Hendel)
Source: Genes (Basel). 2023 Dec 25;15(1):35. doi: 10.3390/genes15010035 (PMC10815873; doi:10.3390/genes15010035)

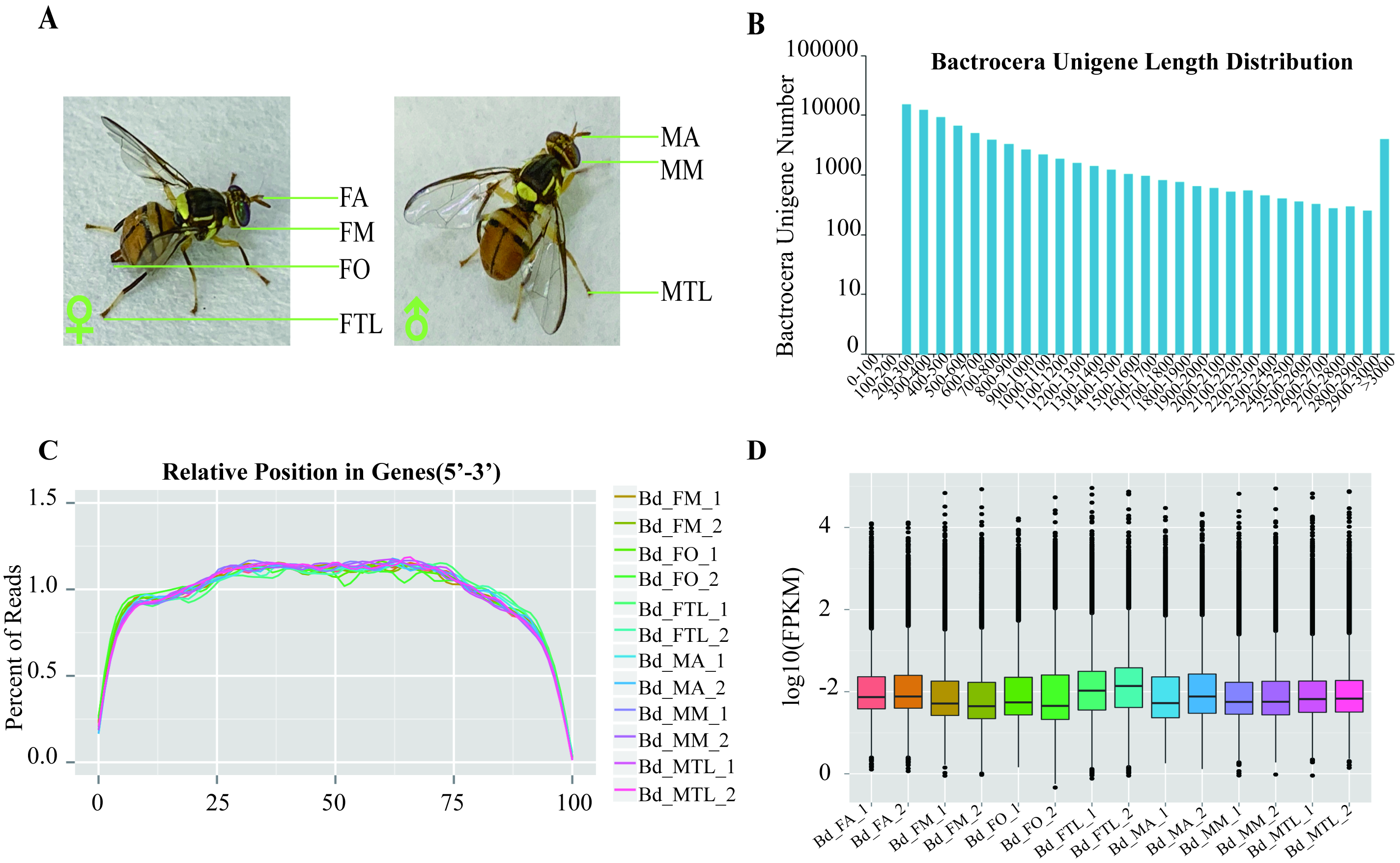

Supplement: Supplementary file 1 [file genes-15-00035-s001.zip › Supplementary/Supplementary Figure S1_Construction of Bactrocera dorsalis Transcriptome.tif]
